# Zn/Fe-Layered Double Hydroxide Composites with Kelp-Derived Biochar for Phosphate Recovery and Reutilization as a Slow-Release Fertilizer

**DOI:** 10.3390/ma19143117

**Published:** 2026-07-20

**Authors:** Jin Yang, Pengcheng Xue, Lu Zhao, Yajuan Luo, Jinfeng Yang, Mengru Wang, Guiying Jiang, Shiliang Liu

**Affiliations:** 1 College of Resources and Environment, Henan Agricultural University, Zhengzhou 450002, China; jyang@henau.edu.cn (J.Y.); 18836622957@163.com (P.X.); 13598361320@163.com (L.Z.); luoyajuan0710@163.com (Y.L.); 13011588490@163.com (J.Y.); m18623860576@163.com (M.W.); jgy9090@126.com (G.J.); 2 State Key Laboratory of High-Efficiency Production of Wheat-Maize Double Cropping, Henan Agricultural University, Zhengzhou 450046, China

**Keywords:** layered double hydroxide, kelp residue biochar, adsorption, phosphorus recovery, slow-release fertilizer

## Abstract

**Highlights:**

- Zn/Fe-layered double hydroxides (Zn/Fe-LDHs) were integrated with kelp residue-derived biochar (KBC) to synthesize Zn/Fe-LDH@KBC composites
- The resulting adsorbent demonstrated a high phosphorus adsorption capacity of 132.52 mg P/g.
- P adsorption mechanisms included electrostatic attraction, surface precipitation, surface complexation, and ion exchange processes.
- P-loaded Zn/Fe-LDH@KBC significantly enhanced pak choi growth and increased soil phosphorus availability.

**Abstract:**

Phosphorus scarcity and inefficient fertilizer utilization highlight the need for sustainable phosphorus recovery and reuse strategies. In this study, a Zn/Fe-layered double hydroxide (Zn/Fe-LDH)-kelp-derived biochar (KBC) composite (Zn/Fe-LDH@0.5KBC) was synthesized via co-precipitation for phosphate capture and subsequent reutilization as a slow-release fertilizer. The incorporation of KBC improved the dispersion of LDH nanosheets and generated a hierarchical porous structure with a specific surface area of 122.13 m^2^/g. As a result, Zn/Fe-LDH@0.5KBC exhibited a high phosphate adsorption capacity of 132.52 mg P/g and reached adsorption equilibrium within 240 min. Kinetic and isotherm analyses indicated that phosphate adsorption was dominated by chemisorption and was best described by the Sips model. Comprehensive scanning electron microscopy (SEM), X-ray diffraction (XRD), Fourier-transform infrared spectroscopy (FTIR), and X-ray photoelectron spectroscopy (XPS) analyses revealed that phosphate removal occurred through synergistic mechanisms, including electrostatic attraction, interlayer anion exchange, surface complexation, and metal phosphate precipitation. The P-loaded composite exhibited diffusion-dominated phosphorus release in soil and significantly enhanced pak choi growth. Compared with the control, labile phosphorus increased from 2.8% to 6.8%, while moderately labile phosphorus increased from 6.3% to 14.1%, indicating improved phosphorus availability. These findings demonstrate an effective strategy for integrating phosphate recovery from wastewater with agricultural reuse and provide insights into the development of multifunctional adsorbent-fertilizer systems for circular phosphorus management.

## 1. Introduction

Phosphorus is an essential nutrient for plant growth and agricultural productivity, playing a fundamental role in crop development, yield formation, and the long-term sustainability of agricultural systems [1,2]. Since the year 2000, global phosphate application has more than doubled in response to rapid population growth and intensified food production, with projections suggesting a further 40% increase by 2060 [3]. Excessive phosphorus inputs, primarily from agricultural runoff and industrial discharges, have substantially increased the risk of eutrophication in water bodies, thereby encouraging excessive algal growth, diminishing dissolved oxygen availability, and threatening ecosystem biodiversity [4,5]. Moreover, phosphorus is a finite, non-renewable resource, with current estimates indicating that global reserves may be depleted within approximately 100 years at the current rate of consumption [6]. In light of these pressing challenges, developing efficient, economically viable, and environmentally sustainable technologies for phosphorus recovery has become increasingly important for conserving phosphorus resources and mitigating environmental degradation.

To date, various strategies for phosphate removal from water have been developed, including biological processes, chemical precipitation, ion exchange, and adsorption [7,8]. Among these approaches, adsorption has been extensively investigated because it combines operational simplicity, economic feasibility, and excellent phosphate removal efficiency [8]. Layered double hydroxides (LDHs) constitute a family of materials that include hydrotalcite and hydrotalcite-like compounds, characterized by the general chemical formula [M^2+^_1−x_M^3+^_x_(OH)_2_]^x+^[A^n−^_x/n_·mH_2_O]^x−^, where M^2+^, M^3+^, and A^n−^ represent divalent and trivalent metal cations and n-valent inorganic or organic anions, respectively [9,10,11]. Recently, LDHs have emerged as promising adsorbents, exhibiting excellent performance in wastewater treatment owing to their unique layered structure, high specific surface area, and strong ion-exchange capacity [4,9,12]. According to previous studies, LDH-based materials exhibit high selectivity toward phosphate and considerable adsorption capacities, which can vary widely depending on composition and experimental conditions, generally varying from several tens to more than 100 mg P/g under environmentally relevant conditions [10,13,14,15]. Moreover, phosphorus-loaded LDHs show potential as slow- or controlled-release phosphorus fertilizers [16,17]. However, the practical application of LDHs is still constrained by several challenges, such as tight packing, poor dispersibility, and difficulties in separation and recovery from aqueous solutions [18,19]. These limitations compromise both the efficiency and the economic feasibility of LDH-based phosphate-removal technologies.

According to previous studies, immobilizing LDHs onto a supporting matrix can significantly enhance their environmental applicability and performance in practical scenarios [18,20]. Biochar, a carbon-rich material produced by pyrolyzing waste biomass under oxygen-limited conditions, is widely regarded as a promising support for LDHs due to its low cost, environmental stability, and additional environmental benefits [2,21]. Biochar features a large specific surface area and a wide variety of surface functional groups, providing abundant reactive sites and interaction interfaces that reduce particle aggregation and improve the chemical stability of LDHs [9,18]. Conversely, LDH modification also enhances the sorption capacity of biochar for oxyanions (e.g., PO_4_^3−^, HPO_4_^2−^, and H_2_PO_4_^−^), owing to the interlayer anion exchangeability and surface complexation properties of LDHs [21]. In this context, various biomass-derived biochars, such as those obtained from wheat straw [1], corn stalk [22], Douglas fir wood [23], macadamia nut shell [24], and pinecone [25], have been investigated as supporting matrices for LDHs to improve phosphate removal and recovery. However, to the best of our knowledge, LDHs/biochar composites fabricated using seaweed-derived biochar as the support have not yet been reported in the literature.

Seaweed, particularly sugar kelp (*Saccharina japonica*), is extensively cultivated in Asia and accounts for 97.38% of global seaweed production [26,27]. Seaweed processing for food and industrial uses generates substantial quantities of kelp residue. Converting this residue into biochar reduces environmental impacts and promotes the recycling and sustainable utilization of marine biomass [26]. Kelp residue-derived biochar (KBC) has demonstrated significant potential for removing a variety of contaminants, including heavy metals, organic dyes, and phosphate [28,29,30,31]. For example, Jung et al. found that biochar derived from waste Undaria pinnatifida and pyrolyzed at 400 °C exhibited excellent phosphate recovery capacity, and the phosphate-laden biochar could serve as an effective phosphorus fertilizer [30]. Additionally, KBC is rich in minerals, making it a promising material for soil amendment [32]. Based on these properties, it is hypothesized that composites formed by combining LDHs with KBC (LDH@KBC) can effectively adsorb phosphorus and enhance the overall adsorption performance, thereby increasing their potential as phosphate fertilizers. To further improve the practical applicability of LDH@KBC composites, Zn^2+^ and Fe^3+^ were selected as the layered metal ions for LDH synthesis in this study. This selection is based on the strong affinity of Fe for phosphorus, which can enhance the adsorption capacity of the composite material [33]. Moreover, both zinc and iron are essential micronutrients for plant growth, broadening the potential agricultural applications of the composites and minimizing the risk of secondary environmental pollution.

In this study, Zn/Fe-layered double hydroxide-engineered kelp-derived biochar composites (Zn/Fe-LDH@KBC) were fabricated through a co-precipitation method and subsequently applied to phosphorus removal and recovery from aqueous solutions. Furthermore, the phosphorus release behavior from phosphorus-loaded adsorbents and their potential application as slow-release fertilizers were systematically investigated. The main objectives of this study were to: (1) examine the impact of preparation conditions on the physicochemical properties and phosphorus adsorption capacity of the composites; (2) investigate how adsorption time, initial phosphate concentration, adsorbent dosage, solution pH, and coexisting ions influence phosphate adsorption by Zn/Fe-LDH@KBC; (3) elucidate the phosphorus adsorption mechanism of Zn/Fe-LDH@KBC through comprehensive kinetic, isotherm, and material characterization analyses; and (4) evaluate the feasibility of using phosphorus-loaded Zn/Fe-LDH@KBC as slow-release phosphate fertilizers for soil amendment purposes.

## 2. Materials and Methods

### 2.1. Chemicals

Kelp residue feedstock was obtained from Guangzhou Shenjingya Agricultural Technology Co., Ltd., Guangzhou, China. Ferric trichloride hexahydrate (FeCl_3_·6H_2_O) and zinc chloride (ZnCl_2_) were from Shanghai Macklin Biochemical Technology Co., Ltd., Shanghai, China. Potassium dihydrogen phosphate (KH_2_PO_4_), sodium hydroxide (NaOH), sodium carbonate (Na_2_CO_3_), hydrochloric acid (HCl), and other reagents came from Sinopharm Chemical Reagent Co., Ltd., Shanghai, China. All chemicals were analytical grade. Deionized water was used throughout all experiments.

### 2.2. Preparation of Zn/Fe LDH-KBC

**Figure 1:**
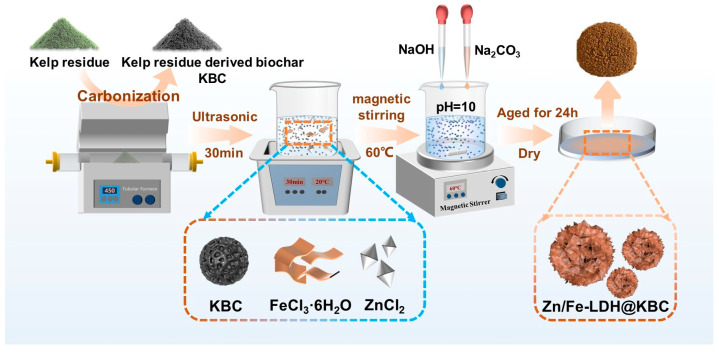
Schematic illustration of the preparation process of Zn/Fe-LDH@KBC.

Kelp residue was rinsed with tap water, then with deionized water, and dried at 80 °C for 24 h. The dried material was ground to 0.25 mm and pyrolyzed in a tubular quartz furnace (GSL 1600X, Hefei Kejing Material Technology Co., Ltd., Hefei, China) at 450 °C for 2 h under a nitrogen flow of 100 mL/min, with a heating rate of 5 °C/min. The resulting biochar (KBC) was washed with 1 M HCl for 12 h and rinsed with deionized water until the pH was neutral. The KBC was then dried at 80 °C, sieved to <0.15 mm, and stored in a sealed container. Zn/Fe-LDH@KBC was synthesized using the co-precipitation method as described by Bian et al. [21]. The preparation process for Zn/Fe-LDH@KBC composites is shown in Figure 1. Briefly, a specified amount of KBC (0.5, 1, 3, or 5 g) was dispersed in 100 mL of an aqueous solution containing 0.02 mol FeCl_3_·6H_2_O and 0.04 mol ZnCl_2_ (nominal Zn:Fe precursor molar ratio = 2:1) by ultrasonication for 30 min. A mixed alkaline solution was prepared by mixing equal volumes of 1.0 M NaOH and 1.0 M Na_2_CO_3_ stock solutions (*v*:*v* = 1:1). The mixed alkaline solution was added dropwise under continuous stirring at 60 °C until the suspension pH reached 10.0. Subsequently, the mixture was stirred for an additional 2 h and aged at 60 °C for 24 h. The resulting Zn/Fe-LDH@KBC precipitate was separated, collected, and repeatedly washed with deionized water until the pH was neutral. The product was dried at 60 °C for 24 h, cooled to room temperature, ground, and stored in a desiccator for further analysis. Based on the KBC content, the composites were labeled as Zn/Fe-LDH@0.5KBC, Zn/Fe-LDH@1.0KBC, Zn/Fe-LDH@3.0KBC, and Zn/Fe-LDH@5.0KBC, respectively. For comparison, pure Zn/Fe-LDH was synthesized using the same procedure without KBC. Additionally, Zn/Fe-LDH@KBC composites with different Zn:Fe molar ratios (2:1, 3:1, and 4:1) were prepared to evaluate the influence of metal ratio on phosphorus adsorption performance.

### 2.3. Batch Adsorption Experiments

Unless otherwise specified, KH_2_PO_4_ solution with a phosphorus concentration of 100 mg P/L (pH 5.0) served as the simulated phosphorus-containing wastewater in batch adsorption experiments. For comparative adsorption tests, 0.025 g of KBC, Zn/Fe-LDH, or various Zn/Fe-LDH@KBC composites was introduced into 25 mL of phosphorus-contaminated solutions at concentrations of 25, 50, and 100 mg P/L (pH 5.0). The mixtures were agitated at 180 rpm for 24 h at 25 °C, then filtered through a 0.45 μm membrane. Residual phosphorus concentrations in the filtrate were quantified using the ammonium molybdate spectrophotometric method at 700 nm with a UV-2600 spectrophotometer (Shimadzu, Japan) [34]. The equilibrium adsorption capacity (qe, mg/g) and removal efficiency (R, %) were calculated according to Equations (1) and (2):

(1) $$q_{e}=\frac{(C_{0}-C_{e})V}{m}$$

(2) $$R=\frac{\left(C_{0}-C_{e}\right)}{C_{0}}\times 100\%$$

where C_0_ and C_e_ denote the initial and equilibrium concentrations of phosphorus in mg P/L, respectively. V (L) represents the volume of the phosphorus solution, and m (g) refers to the mass of KBC, Zn/Fe-LDH, or Zn/Fe-LDH@KBC composites.

This approach was employed to assess the effects of initial solution pH (2.0–9.0), adsorbent dosage (0.4–2.0 g/L), adsorption time (5–1440 min), initial phosphorus concentration (20–300 mg/L), and coexisting anions (Cl^−^, NO_3_^−^, SO_4_^2−^, HCO_3_^−^, and CO_3_^2−^) on phosphorus adsorption. Furthermore, adsorption kinetic models (pseudo-first-order, pseudo-second-order, Elovich, and intra-particle diffusion) and isotherm models (Langmuir, Freundlich, Sips, and Temkin) were utilized to analyze the phosphorus adsorption behavior of Zn/Fe-LDH@KBC [35,36]. The relevant equations and parameters for these models are presented in Text S1.

### 2.4. Material Characterization Methods

The crystalline structures of Zn/Fe-LDH@KBC before and after phosphorus adsorption were characterized using an X-ray diffractometer (XRD, Rigaku SmartLab SE, Japan) equipped with Cu Kα radiation (λ = 1.5406 Å). XRD patterns were collected over a 2θ range of 10–80° at a scanning rate of 5° min^−1^. Functional groups were identified by Fourier-transform infrared spectroscopy (FTIR, Thermo Fisher Nicolet iS20, USA) over the range of 4000–400 cm^−1^ using the KBr pellet method. Surface morphology and elemental composition were examined using field-emission scanning electron microscopy (SEM, Carl Zeiss Sigma 360, Germany) coupled with energy-dispersive X-ray spectroscopy (EDS, Bruker Xflash 6/60, Germany). Prior to SEM observation, the dried samples were mounted on conductive adhesive tape and sputter-coated with gold. Nitrogen adsorption–desorption isotherms were measured using a Brunauer–Emmett–Teller (BET) analyzer (BELSORP-mini, BYK, Japan). Before analysis, samples were degassed under vacuum at 120 °C for 6 h. The specific surface area was calculated using the BET method, and pore size distribution was obtained using the BJH method. Elemental composition and surface chemical states of Zn/Fe-LDH@KBC and Zn/Fe-LDH@KBC-P were analyzed by X-ray photoelectron spectroscopy (XPS, EscaLab250Xi, Thermo Fisher Scientific, USA) with an Al Kα X-ray source (1486.6 eV). The binding energies were calibrated using the C 1s peak at 284.8 eV. The actual Zn/Fe molar ratios of the synthesized composites were determined by inductively coupled plasma optical emission spectrometry (ICP-OES) after acid digestion using an HNO_3_/HClO_4_ (3:1, *v*/*v*) mixture.

### 2.5. Phosphorus Release Behavior in the Zn/Fe-LDH@KBC-P Amended Soils

Saturated adsorbents were prepared by immersing 2.0 g of Zn/Fe-LDH@0.5KBC in 1 L of 500 mg/L phosphate solution at pH 3.0. After reaching equilibrium, the materials were rinsed with ultrapure water, dried at 105 °C for 24 h, and designated as Zn/Fe-LDH@0.5KBC-P. For the release experiment, 1.0 g of Zn/Fe-LDH@0.5KBC-P was thoroughly mixed with 19.0 g of phosphorus-deficient soil. The mixture was transferred to a glass funnel lined with double-layer filter paper to prevent soil loss and facilitate clear leachate collection. Soil saturation was achieved from the bottom of the funnel by capillary action. During leaching, 50 mL of ultrapure water was applied every 24 h using a rubber bulb dropper. Two layers of filter paper were placed on the soil surface to ensure uniform water infiltration. Leachate samples were collected at each interval, filtered through a 0.45 μm membrane, and analyzed for phosphorus content. The corresponding leachate volume was recorded. Phosphorus release kinetics were fitted to the zero-order, pseudo-first-order, Elovich, and Higuchi models [37,38]. The corresponding equations and model parameters are detailed in Text S2.

### 2.6. Pot Experiments

Phosphorus-deficient soil samples were collected from Yuanyang County, Henan Province, China (35.11° N, 113.95° E). The soil is classified as fluvo-aquic, characterized by alluvial origin, moderate fertility, and widespread cultivation. This soil type is representative of the dominant arable soils in the North China Plain. The basic physicochemical properties of the soil are presented in Table S1. Zn/Fe-LDH@KBC-P was applied as a soil amendment at dosages of 0.1%, 0.5%, and 1% (*w*/*w*), with diammonium phosphate (DAP) serving as a positive control. Five treatments were established: non-amended soil (CK), soil amended with DAP (with phosphorus content equivalent to the 0.1% phosphorus-loaded composite), and soils amended with 0.1%, 0.5%, and 1% Zn/Fe-LDH@KBC-P (designated as LBP-0.1, LBP-0.5, and LBP-1.0, respectively). Each treatment was replicated five times using plastic pots (8 cm diameter × 7 cm height), each containing 250 g of soil. Ten pak choi (*Brassica chinensis* L.) seeds were sown per pot. The pots were maintained in a greenhouse under a 16 h light and 8 h dark photoperiod. After germination, seedlings were thinned to two plants per pot. Irrigation was performed every two days to maintain soil moisture. Soil and plant samples were collected 21 days after planting for subsequent analysis.

### 2.7. Soil Sampling and Plant Sampling Measurements

Soil samples were air-dried, sieved, and analyzed for total phosphorus (TP), available phosphorus (AP), and inorganic phosphorus fractions. TP was extracted using H_2_SO_4_-HClO_4_ digestion, and AP was extracted with 0.5 M NaHCO_3_ [39]. Inorganic phosphorus fractions, including H_2_O-P, NaHCO_3_-Pi, NaOH-Pi, dilute HCl-Pi, and Residual-P, were extracted using the modified Hedley P fractionation method [40]. Phosphorus concentrations in all extracts were determined spectrophotometrically using the molybdenum blue method [34]. Pak choi samples were oven-dried at 105 °C for 30 min, then dried at 80 °C until reaching a constant weight, and subsequently the dry weights of the shoot and root parts were recorded.

### 2.8. Data Analysis and Processing

All experiments were performed with three independent replicates, and the data are expressed as mean ± standard error (SE). Statistical analyses were carried out using SPSS 22.0 software. Significant differences among treatments were evaluated by one-way analysis of variance (ANOVA). All figures were generated using Origin 2024 software.

## 3. Results and Discussion

### 3.1. Comparison of Various Materials

**Figure 2:**
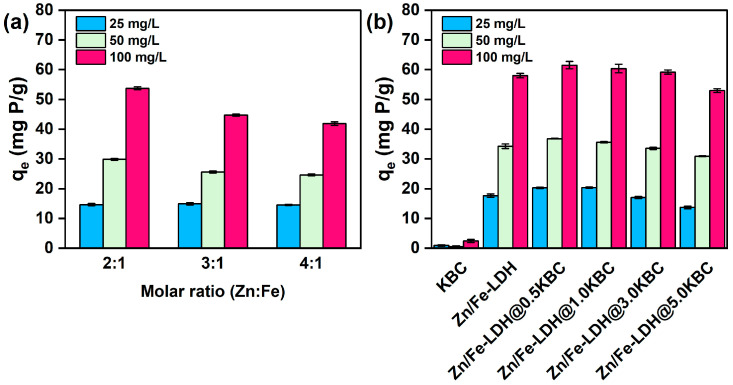
(**a**) Phosphorus adsorption by Zn/Fe-LDH@KBC at different metal molar ratios and (**b**) Phosphorus adsorption by Zn/Fe-LDH@KBC with different KBC content.

The M^2+^/M^3+^ molar ratio is a critical synthesis parameter influencing the adsorption properties of LDHs and LDH/biochar composites [41]. As shown in Figure 2a, Zn/Fe-LDH@KBC prepared with a nominal Zn/Fe precursor ratio of 2:1 exhibited the highest phosphate adsorption capacity, particularly at elevated phosphate concentrations. This observation is consistent with previous studies demonstrating that the M^2+^/M^3+^ ratio governs the layer charge density, interlayer spacing, morphology, and anion-exchange behavior of LDH materials, thereby strongly affecting their adsorption performance [42]. For example, Mg-Al LDH-functionalized hydrochar prepared at an Mg/Al ratio of 2:1 exhibited the highest phosphate removal due to its favorable layer charge density and enlarged interlayer spacing [41]. Similarly, Mg-La LDH synthesized at a 2:1 ratio showed superior phosphate adsorption compared with samples prepared at higher Mg/La ratios, whereas excessive Mg contents caused structural distortion and reduced anion-exchange capability [43]. To determine the actual elemental composition, ICP-OES analysis was conducted for composites synthesized with nominal Zn/Fe precursor ratios of 2:1, 3:1, and 4:1. As summarized in Table S2, the corresponding actual Zn/Fe molar ratios were 2.76, 3.69, and 5.84, respectively. Although these values were somewhat higher than the nominal precursor ratios, likely owing to the relatively lower incorporation efficiency and partial loss of Fe species during co-precipitation and washing processes, the measured ratios exhibited the same increasing trend, indicating that the precursor composition effectively regulated the Zn/Fe composition of the resulting composites. At low phosphate concentrations, sufficient adsorption sites were available on all composites, resulting in only minor differences in adsorption capacities. However, as phosphate concentration increased, adsorption sites gradually approached saturation, making differences in active-site density and phosphate affinity more pronounced. Excessive Zn enrichment at higher Zn/Fe ratios may reduce the abundance of Fe-related active sites responsible for phosphate surface complexation and precipitation, thereby weakening phosphate affinity and leading to inferior adsorption performance. Consequently, the composite prepared with a nominal Zn/Fe precursor ratio of 2:1 (actual Zn/Fe ratio ≈ 2.76) was selected for subsequent studies. The impact of the Zn/Fe-LDH to KBC mixing ratio on phosphorus adsorption is presented in Figure 2b. At 100 mg P/L, pristine Zn/Fe-LDH achieves a maximum phosphorus adsorption capacity of 58.01 mg P/g, confirming its effectiveness in phosphorus removal. Incorporation of KBC initially increases the adsorption capacity of Zn/Fe-LDH@KBC, but it subsequently decreases as the KBC content increases. Among the tested composites, Zn/Fe-LDH@0.5KBC exhibited the highest phosphorus adsorption capacity and was therefore selected for subsequent characterization and mechanistic studies. This trend indicates that KBC enhances LDH adsorption performance, likely by reducing LDH layer aggregation and stacking [44]. In contrast, pure KBC demonstrates negligible phosphorus adsorption, accounting for the inferior performance of Zn/Fe-LDH@5.0KBC compared to pristine Zn/Fe-LDH. Therefore, Zn/Fe-LDH@0.5KBC was selected for further experiments.

### 3.2. Adsorption Performance of Zn/Fe-LDH@0.5KBC

#### 3.2.1. Effect of Initial pH

**Figure 3:**
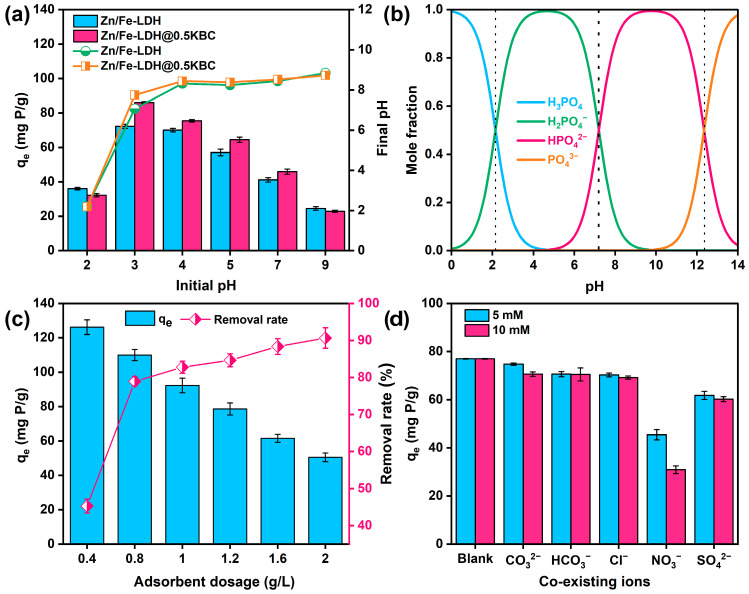
Adsorption performance of phosphorus on the adsorbent. (**a**) Effect of initial pH on the final pH and phosphorus adsorption capacity of Zn/Fe-LDH and Zn/Fe-LDH@0.5KBC; (**b**) speciation of phosphorus at different pH; (**c**) effect of adsorbent dosage; (**d**) effect of coexisting anions.

The pH significantly influences both the adsorbent’s surface properties and the speciation of phosphate in solution, thereby affecting the dominant adsorption mechanism and overall adsorption performance [45]. As shown in Figure 3a, increasing the initial pH from 2.0 to 9.0 led to a sharp rise followed by a gradual decline in the phosphorus adsorption capacity of Zn/Fe-LDH@0.5KBC. The layered structure of Zn/Fe-LDHs dissolved at pH 2.0, resulting in a marked reduction in adsorption capacity. The maximum adsorption capacity of 86.02 mg P/g was observed at pH 3.0. At pH 9.0, Zn/Fe-LDH@0.5KBC lost more than 70% of its phosphorus adsorption capacity. The pH-dependent trend for Zn/Fe-LDH mirrored that of Zn/Fe-LDH@0.5KBC. Compared to pristine Zn/Fe-LDH, Zn/Fe-LDH@0.5KBC demonstrated significantly enhanced phosphorus adsorption capacity and removal efficiency across all pH levels, except at pH 2.0 and 9.0.

As shown in Figure 3b, phosphate speciation in solution strongly depends on pH. H_2_PO_4_^−^ (pKa > 2.16) is the dominant phosphate species under acidic to near-neutral conditions, whereas HPO_4_^2−^ (7.20 < pKa < 12.35) becomes predominant at higher pH values [46]. The zeta potential results showed that the point of zero charge (pHzpc) of Zn/Fe-LDH@0.5KBC was 7.32 (Figure S1), indicating that the composite surface was positively charged at pH values below 7.32. Therefore, within the pH range of 3.0–7.0, electrostatic attraction between the positively charged adsorbent surface and negatively charged phosphate species favored phosphate adsorption [10]. When the solution pH exceeded the pHzpc, surface deprotonation resulted in a negatively charged surface, weakening electrostatic attraction and increasing electrostatic repulsion toward phosphate species. Meanwhile, the transformation of phosphate species from H_2_PO_4_^−^ to HPO_4_^2−^ may further reduce adsorption due to stronger electrostatic repulsion with the negatively charged surface [22]. The relatively high adsorption capacity observed at pH 3.0 may also be associated with enhanced interactions between phosphate and Zn/Fe-containing active sites under acidic conditions. However, this interpretation should be considered together with the subsequent FTIR, XPS, and SEM analyses, which provide further evidence for surface complexation and phosphate-containing precipitates after adsorption. In addition, the final pH values stabilized at approximately 8.0–9.0 for most initial pH conditions, except at pH 2.0 (Figure 3a). This pH buffering behavior suggests that proton consumption, surface hydroxyl reactions, and ligand exchange may also participate in phosphate adsorption [47].

#### 3.2.2. Effect of Adsorbent Dosage

Figure 3c presents the effect of adsorbent dosage on phosphorus adsorption. Increasing the Zn/Fe-LDH@0.5KBC dosage from 0.4 g/L to 2.0 g/L resulted in a gradual decrease in phosphorus adsorption capacity, while the removal rate increased substantially. For example, at 2.0 g/L, the phosphate removal rate reached 90.67%, although the adsorption capacity decreased to 50.54 mg P/g. Higher adsorbent dosages provide more active sites for phosphorus adsorption, thereby enhancing the removal rate; however, this also leads to underutilization of the adsorbent, reducing adsorption capacity per unit mass [47]. Considering the trade-off between adsorption capacity and removal efficiency, 1.0 g/L was identified as optimal and used in subsequent experiments.

#### 3.2.3. Effect of Coexisting Ions

The presence of various inorganic ions in wastewater can influence the phosphorus removal efficiency of adsorbents. To facilitate comparison among coexisting anions with different molecular weights, their concentrations were expressed on a molar basis (mM). The coexisting anions were tested at concentrations of 5 and 10 mM, corresponding to approximately 300 and 600 mg/L for CO_3_^2−^, 305 and 610 mg/L for HCO_3_^−^, 177 and 355 mg/L for Cl^−^, 310 and 620 mg/L for NO_3_^−^, and 480 and 961 mg/L for SO_4_^2−^, respectively (Table S3). As illustrated in Figure 3d, the introduction of coexisting inorganic anions (CO_3_^2−^, HCO_3_^−^, Cl^−^, NO_3_^−^, and SO_4_^2−^; each added separately at pH 5) resulted in different effects on the phosphorus adsorption capacity of Zn/Fe-LDH@0.5KBC. The order of interference was NO_3_^−^ > SO_4_^2−^ > Cl^−^ > HCO_3_^−^ > CO_3_^2−^. The addition of 5 mM and 10 mM NO_3_^−^ resulted in significant decreases in the adsorption capacity of 40.92% and 59.81%, respectively. This reduction is likely due to competitive ion exchange between NO_3_^−^ and H_2_PO_4_^−^ within the interlayer anion sites of Zn/Fe-LDH@0.5KBC under acidic conditions [22,47]. The presence of SO_4_^2−^ moderately reduced the phosphorus adsorption capacity by approximately 20%. In contrast, Cl^−^, HCO_3_^−^, and CO_3_^2−^ had minimal effects (<10% reduction), likely due to their weaker competitive ability for adsorption sites compared to phosphate ions.

#### 3.2.4. Adsorption Kinetics and Isotherms

**Figure 4:**
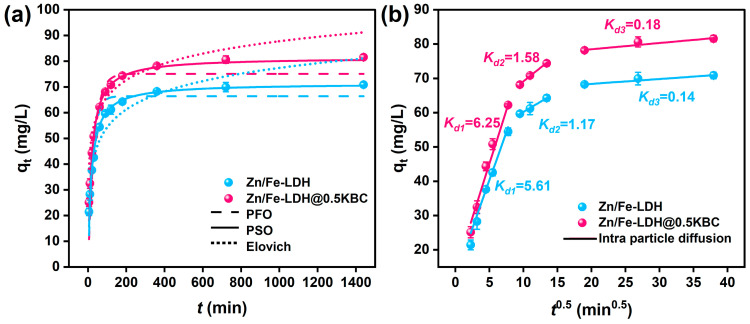
Adsorption kinetics of phosphorus adsorption onto Zn/Fe-LDH and Zn/Fe-LDH@0.5KBC. (**a**) Fitting results using PFO, PSO and Elovich models; (**b**) fitting results using intra particle diffusion model.

Figure 4a demonstrates that most phosphorus adsorption occurred within the first 60 min, during which Zn/Fe-LDH and Zn/Fe-LDH@0.5KBC achieved 76.85% and 76.22% of their equilibrium adsorption capacities, respectively. The rapid initial uptake can be explained by the abundant accessible active sites available on the adsorbent surface. As contact time increased, these sites became progressively occupied, approaching apparent equilibrium. To better characterize the adsorption kinetics, the experimental data were fitted to four commonly used kinetic models, namely the pseudo-first-order (PFO), pseudo-second-order (PSO), Elovich, and intraparticle diffusion models. The corresponding model parameters are presented in Table 1. Both Zn/Fe-LDH and Zn/Fe-LDH@0.5KBC exhibited higher coefficients of determination (R^2^) for PSO kinetics (0.995 and 0.991, respectively) compared to PFO (0.961 and 0.844), suggesting that chemisorption dominates the adsorption process [48]. To further investigate the rate-controlling steps, the intraparticle diffusion model was applied. As illustrated in Figure 4b, the model showed a good fit for both Zn/Fe-LDH and Zn/Fe-LDH@0.5KBC. The fitting curve could be divided into three linear segments, none of which passed through the origin. This indicates that the adsorption of phosphate onto the two adsorbents involves three sequential stages: (1) Rapid surface adsorption via external diffusion; (2) Diffusion of ions into internal mesopores and micropores; and (3) Gradual occupation of adsorption sites until equilibrium is reached [1]. The fact that none of the fitted lines intersected the origin suggests that intraparticle diffusion was not the only rate-controlling mechanism, and that the adsorption process was jointly governed by multiple mass-transfer steps [49].

**Figure 5:**
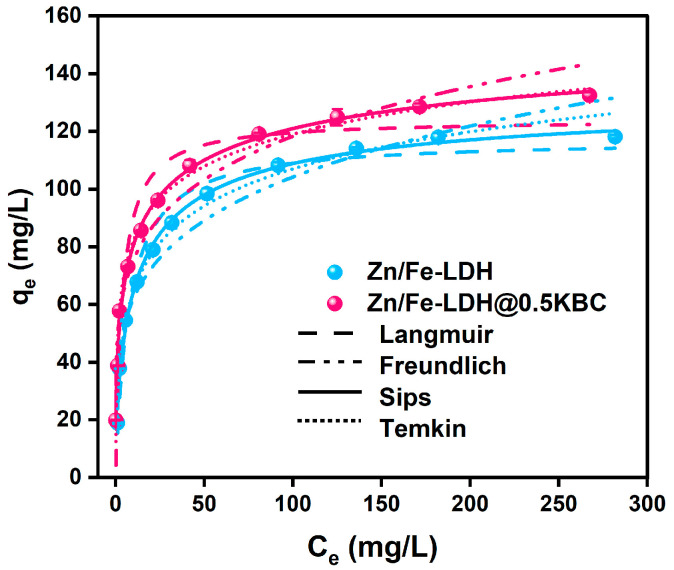
Isotherm fitting results of phosphorus adsorption onto Zn/Fe-LDH and Zn/Fe-LDH@0.5KBC.

Figure 5 depicts the relationship between phosphorus adsorption capacity and equilibrium concentration for both adsorbents. As phosphorus concentration increases, adsorption capacity rises until saturation is reached. Zn/Fe-LDH and Zn/Fe-LDH@0.5KBC exhibit maximum experimental phosphorus adsorption capacities of 118.01 mg P/g and 132.52 mg P/g, respectively. To characterize the adsorption behavior, the experimental data were fitted to the Langmuir, Freundlich, Sips, and Temkin isotherm models. As shown in Table 2, all four models yielded R^2^ values above 0.90, indicating strong agreement with the experimental data. The Sips model provided the best fit (R^2^ > 0.99), suggesting that phosphate ions are adsorbed onto Zn/Fe-LDH and Zn/Fe-LDH@0.5KBC via both monolayer and multilayer mechanisms [50]. At low adsorbate concentrations, adsorption behavior follows the Freundlich isotherm, indicating multilayer adsorption on heterogeneous surfaces, while at high concentrations, it aligns with the Langmuir isotherm, reflecting monolayer adsorption on homogeneous sites [51]. Furthermore, the Temkin model also demonstrated high fitting coefficients (R^2^ = 0.987 and 0.986 for Zn/Fe-LDH and Zn/Fe-LDH@0.5KBC, respectively), indicating that phosphate ions can interact with the adsorbents via electrostatic or other physical forces [52].

The Langmuir isotherm model estimated a maximum phosphate adsorption capacity of 124.11 mg P/g for Zn/Fe-LDH@0.5KBC, exceeding the value obtained for pristine Zn/Fe-LDH (117.17 mg P/g). These results suggest that the incorporation of KBC significantly improves the phosphate adsorption performance of the composite. It is noted that the Langmuir-derived capacity is slightly lower than the experimentally observed value (132.52 mg P/g), which can be attributed to the heterogeneous surface and the contribution of multiple adsorption mechanisms beyond the monolayer assumption. Notably, the Sips model yielded a higher theoretical adsorption capacity (163.49 mg P/g), further supporting the heterogeneous nature of the adsorption process [51]. To further evaluate the adsorption performance, Langmuir-derived q_max_ values were compared with those of previously reported adsorbents. As summarized in Table S4, Zn/Fe-LDH@0.5KBC exhibited a superior or comparable adsorption capacity relative to a wide range of LDH/biochar composite materials, highlighting its competitive performance for phosphorus removal and recovery.

### 3.3. Phosphorus Adsorption Mechanism

As depicted in Figure S1, the pHzpc values of Zn/Fe-LDH and Zn/Fe-LDH@0.5KBC were determined to be 8.81 and 7.32, respectively, indicating that the incorporation of negatively charged KBC effectively reduced the surface charge of the composite. Notably, LDH inherently possesses positively charged layers, while KBC exhibited a strongly negative surface charge (−21.9 mV at pH 7), suggesting the presence of abundant oxygen-containing functional groups on the biochar surface [53]. Based on these results, the Zn/Fe-LDH@0.5KBC surface remains positively charged under acidic to near-neutral conditions, facilitating the electrostatic attraction of phosphate species during the initial adsorption stage.

**Figure 6:**
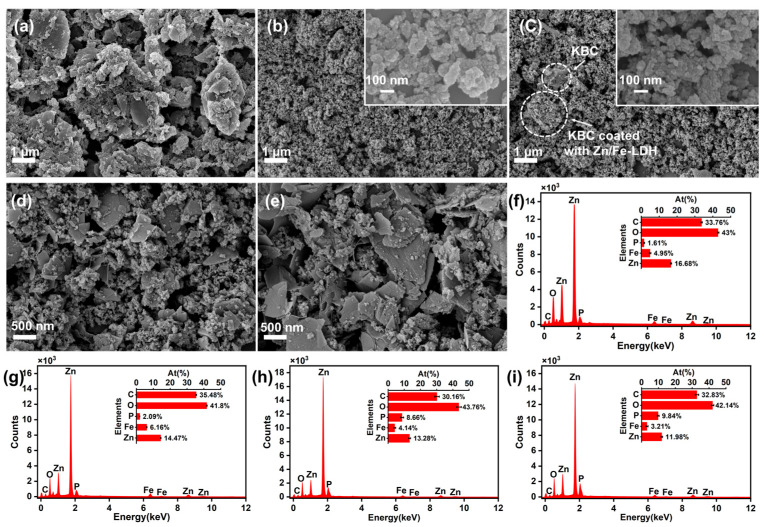
SEM images of KBC, Zn/Fe-LDH, and Zn/Fe-LDH@0.5KBC before and after phosphorus adsorption, together with the corresponding EDS spectra of LDH-based samples. (**a**) KBC; (**b**) Zn/Fe-LDH; (**c**) Zn/Fe-LDH@0.5KBC; (**d**) Zn/Fe-LDH after adsorption; (**e**) Zn/Fe-LDH@0.5KBC after adsorption; (**f**–**i**) EDS spectra corresponding to (**b**–**e**), respectively.

As shown in Figure 6a, KBC exhibited a rough and irregular carbon framework with a relatively loose structure, which could provide anchoring sites for the growth of LDH nanoparticles. In contrast, Zn/Fe-LDH showed a highly aggregated morphology composed of densely stacked nanosheets, indicating obvious restacking during synthesis (Figure 6b). After the introduction of KBC, Zn/Fe-LDH@0.5KBC displayed a more open and homogeneous structure, with LDH nanoparticles dispersed on the KBC surface (Figure 6c). This result suggests that KBC acted as a supporting matrix and helped suppress the aggregation of LDH nanosheets. The magnified inset images in Figure 6b,c further show that Zn/Fe-LDH consisted of irregular plate-like nanoparticles with sizes of approximately 50–200 nm. The rough particle surface may be related to incomplete crystallization during the co-precipitation synthesis of LDHs [1,21]. Compared with pristine Zn/Fe-LDH, Zn/Fe-LDH@0.5KBC exhibited a smoother surface and more uniformly dispersed particles, indicating that KBC facilitated LDH dispersion and reduced nanosheet restacking. Additional SEM images at different magnifications (Figure S2) further support this observation. To further verify the spatial distribution of the composite components, SEM-EDS elemental mapping was performed for Zn/Fe-LDH@0.5KBC before phosphate adsorption (Figure S4). The C, O, Zn, and Fe signals were distributed throughout the composite particles, confirming the successful anchoring of Zn/Fe-LDH on the KBC framework. These results provide direct elemental evidence for the formation of the Zn/Fe-LDH@KBC composite.

To further evaluate the pore structure, N_2_ adsorption–desorption measurements were conducted. As shown in Figure S3, the N_2_ adsorption–desorption isotherms of both samples were identified as type IV with distinct hysteresis loops based on the IUPAC classification, confirming their mesoporous characteristics [1,54]. The BET surface area increased from 94.41 m^2^/g for Zn/Fe-LDH to 122.13 m^2^/g for Zn/Fe-LDH@0.5KBC, while the total pore volume increased from 0.64 cm^3^/g to 0.71 cm^3^/g (Table S5). The average pore diameters are 27.25 nm and 23.55 nm, respectively, confirming the dominance of mesoporous structures. The pore size distribution shows peaks around 1–2 nm and 30–35 nm, indicating the coexistence of micro- and mesopores [1]. The enhanced surface area and pore volume can be attributed to the presence of KBC, which suppresses LDH restacking and creates additional pore channels, thereby facilitating phosphate diffusion and mass transfer [55].

As illustrated in Figure 6d,e, obvious morphological changes were observed after phosphate adsorption. The original aggregated structures of Zn/Fe-LDH and Zn/Fe-LDH@0.5KBC became less distinct, and newly formed plate-like or blocky crystalline structures appeared on the adsorbent surface. These newly generated structures were larger and more regular than the original LDH nanoparticles, suggesting the formation of secondary phosphate-containing phases during adsorption. This phenomenon may be associated with the interaction between phosphate species and Zn/Fe-containing active sites, leading to surface precipitation and/or surface complexation [10,56]. The EDS spectra further confirmed phosphate uptake after adsorption (Figure 6f–i). A distinct P signal appeared in all P-loaded samples, indicating successful phosphate adsorption. Notably, Zn/Fe-LDH@0.5KBC showed a higher P atomic percentage than pristine Zn/Fe-LDH, which is consistent with its superior adsorption performance. In addition, the SEM-EDS mapping results of Zn/Fe-LDH@0.5KBC before and after phosphate adsorption (Figure S4) provided more direct spatial evidence. Before adsorption, C, O, Zn, and Fe were distributed throughout the composite particles, confirming the anchoring of Zn/Fe-LDH on the KBC framework. After adsorption, the P signal became clearly visible and was mainly distributed in the Zn/Fe-rich regions, indicating that phosphate was preferentially retained at Zn/Fe-containing active sites. Together, these SEM, EDS, and mapping results support that phosphate removal by Zn/Fe-LDH@0.5KBC involved Zn/Fe-associated surface reactions, including surface complexation and phosphate-containing precipitate formation.

**Figure 7:**
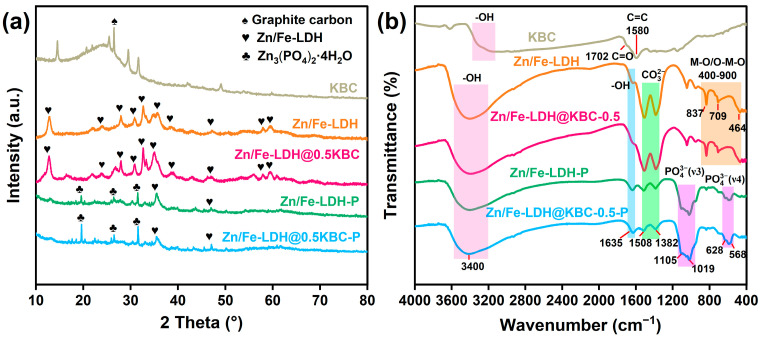
XRD (**a**) and FTIR (**b**) spectra of KBC, Zn/Fe-LDH and Zn/Fe-LDH@0.5KBC before and after phosphorus adsorption.

Figure 7a illustrates the XRD patterns of Zn/Fe-LDH and Zn/Fe-LDH@0.5KBC obtained before and after phosphate adsorption. KBC displayed a broad diffraction peak, indicating an amorphous structure, along with a sharp peak at 26.53° corresponding to the (002) plane of graphite carbon [57]. Both materials exhibited characteristic diffraction peaks at 12.80°, 23.90°, 27.94°, 30.86°, 32.64°, 35.00°, 38.54°, and 59.54°, which were assigned to the crystalline planes (003), (006), (110), (009), (101), (116), (015), and (110) of the carbonate-intercalated LDH phase [58,59]. Compared to Zn/Fe-LDH, Zn/Fe-LDH@0.5KBC exhibited stronger diffraction peaks, indicating improved crystallinity, which aligns with the SEM observations. After phosphorus adsorption, the LDH-related peaks in both samples weakened or disappeared, indicating structural disruption during adsorption. However, several new sharp peaks appeared in the XRD patterns of the phosphorus-loaded samples, matching the diffraction pattern of hopeite (Zn_3_(PO_4_)_2_·4H_2_O, PDF#01-0975). This indicates that phosphate reacted with free Zn^2+^ ions to form insoluble phosphate compounds [60]. This observation was further supported by the presence of thin sheet-like crystal structures in the SEM images. Both Zn/Fe-LDH and Zn/Fe-LDH@0.5KBC reached their maximum phosphate adsorption capacities at pH 3.0, which was likely associated with enhanced Zn^2+^ release under acidic conditions. Collectively, these results indicate that surface precipitation represents one of the dominant mechanisms responsible for phosphate adsorption by both materials [61]. Notably, the Zn/Fe-LDH@0.5KBC sample exhibited a more intense diffraction peak corresponding to hopeite compared to Zn/Fe-LDH, confirming its higher phosphorus adsorption capacity.

Figure 7b compares the FTIR spectra of Zn/Fe-LDH and Zn/Fe-LDH@0.5KBC before and after phosphate adsorption. For KBC, the peaks at 3244, 1702, and 1580 cm^−1^ correspond to the stretching vibrations of -OH, carboxyl C=O, and aromatic C=C functional groups, respectively [35]. In the FTIR spectra of Zn/Fe-LDH and Zn/Fe-LDH@0.5KBC, the strong absorption band at 3414 cm^−1^ was attributed to the stretching vibration of -OH groups within the hydroxide layers, whereas the peak at 1635 cm^−1^ is assigned to the H-O-H bending vibration of adsorbed water molecules [5]. The split peaks at 1508 and 1382 cm^−1^ are characteristic of carbonate anions (CO_3_^2−^) located in the interlayer region, which were introduced during synthesis using Na_2_CO_3_ [22]. Additionally, the broad absorption bands observed between 400 and 900 cm^−1^ are primarily assigned to the lattice vibrations of M-O and O-M-O bonds (where M represents Zn^2+^ or Fe^3+^), which are typical of layered double hydroxides [62]. Consistent with SEM observations, the surface coverage by Zn/Fe-LDH hindered the clear detection of characteristic KBC peaks in the composite.

Following phosphate adsorption, two additional characteristic absorption bands appeared at 1019–1105 cm^−1^ and 568–628 cm^−1^, corresponding to the P-O stretching (ν_3_) and O-P-O bending (ν_4_) vibrational modes, respectively [20,63]. This confirms the successful adsorption of phosphate by both two adsorbents. Notably, Zn/Fe-LDH@0.5KBC-P exhibited stronger peak intensities compared to Zn/Fe-LDH-P, indicating a higher phosphorus uptake capacity of Zn/Fe-LDH@0.5KBC. The phosphorus-loaded materials also showed an enhanced peak at 1635 cm^−1^, which can be attributed to the presence of crystalline water in Zn_3_(PO_4_)_2_·4H_2_O, further supporting the XRD results. Moreover, the -OH stretching peak at 3414 cm^−1^ significantly weakened and broadened after phosphorus adsorption, suggesting that hydroxyl groups participated in the adsorption process through ligand exchange [1,64]. Further evidence for this interpretation is provided by the increase in the final solution pH following adsorption. The CO_3_^2−^-related peaks at 1508 and 1382 cm^−1^ also weakened considerably, indicating that interlayer carbonate ions were partially replaced by phosphate via anion exchange [5,60]. Additionally, the intensity of the M-O and M-O-M lattice vibration bands in the 400–900 cm^−1^ range decreased after phosphorus adsorption. This decrease is likely due to the formation of Zn-O-P or Fe-O-P surface complexes through coordination interactions [5,35]. Taken together, these findings suggest that the phosphorus adsorption mechanism involves surface complexation and interlayer anion exchange.

**Figure 8:**
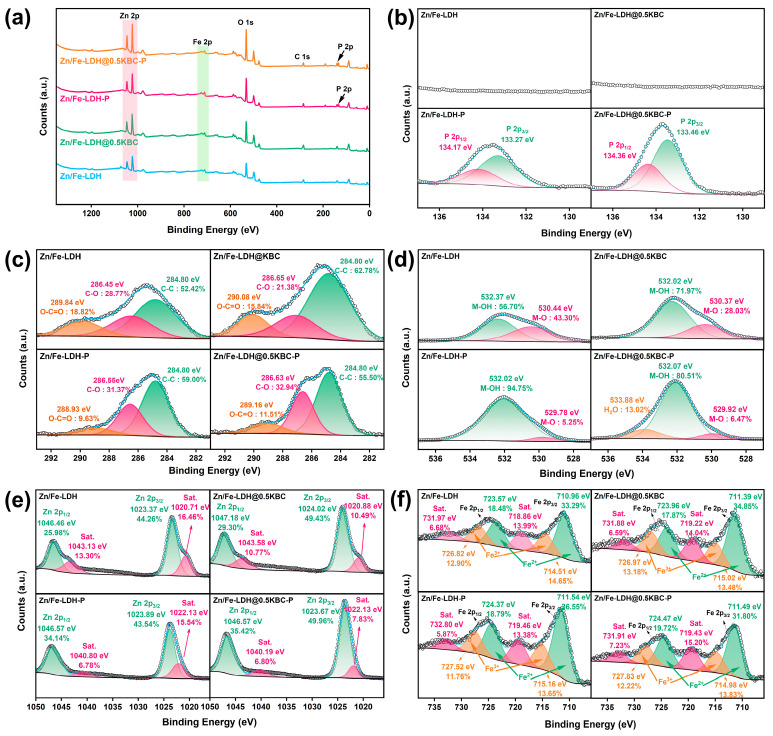
XPS spectrum of Zn/Fe-LDH and Zn/Fe-LDH@0.5KBC before and after phosphorus adsorption. Full survey XPS spectrum (**a**); high resolution XPS spectrum of P 2p (**b**), C 1s (**c**), O 1s (**d**), Zn 2p (**e**), and Fe2p (**f**).

XPS analysis was conducted to further elucidate the phosphorus adsorption mechanisms of Zn/Fe-LDH and Zn/Fe-LDH@0.5KBC. Figure 8a presents the XPS survey spectra for Zn 2p, Fe 2p, O 1s, and C 1s, confirming the presence of Zn, Fe, O, and C in both materials. The incorporation of KBC enhances the carbon content, resulting in a more prominent C 1s peak in Zn/Fe-LDH@0.5KBC (Figure 8a,c). Following phosphate adsorption, a characteristic P 2p peak appeared in the XPS survey spectra of both Zn/Fe-LDH and Zn/Fe-LDH@0.5KBC, providing direct evidence for the successful adsorption of phosphate onto the adsorbent surfaces. The high-resolution P 2p spectrum of Zn/Fe-LDH@0.5KBC-P could be deconvoluted into two spin–orbit splitting peaks at 133.46 eV and 134.36 eV, corresponding to P 2p_3/2_ and P 2p_1/2_, respectively [56] (Figure 8b). Relative to the P 2p binding energy of purified KH_2_PO_4_ (134.0 eV), a 0.54 eV negative shift was observed after adsorption, indicating the formation of Fe-O-P and Zn-O-P inner-sphere complexes through Lewis acid–base interactions [46,65]. The P 2p spectra of Zn/Fe-LDH were similar to those of Zn/Fe-LDH@0.5KBC; however, the lower peak intensity indicates a weaker phosphorus adsorption capacity. The C 1s spectra of Zn/Fe-LDH (Zn/Fe-LDH@0.5KBC) were deconvoluted into three distinct peaks at 284.80 eV, 286.45 eV (286.65 eV), and 289.45 eV (290.08 eV), corresponding to C-C, C-O, and O-C=O bonding configurations, respectively [46]. After phosphorus adsorption, the relative area ratios of the O-C=O component decreased by 9.19% and 9.87% for Zn/Fe-LDH and Zn/Fe-LDH@0.5KBC, respectively, further supporting the involvement of CO_3_^2−^ in phosphorus adsorption via interlayer anion exchange, consistent with FTIR analysis (Figure 8c) [5]. The O 1s spectra before and after phosphate adsorption were fitted into two peaks corresponding to M-O and M-OH oxygen species [46]. After phosphorus adsorption, the relative proportions of M-O decreased by 38.05% and 15.01% for Zn/Fe-LDH and Zn/Fe-LDH@0.5KBC, respectively, while the area ratio of M-OH increased (Figure 8d). This is primarily due to the replacement of hydroxyl groups coordinated to metal ions on the LDH surface by phosphate oxygen atoms, forming inner-surface P-O-M complexes [66]. This ligand exchange leads to local structural reconstruction on the LDH surface, with M-O (lattice oxygen) bonds breaking and new M-OH groups forming through protonation. Interestingly, a distinct H_2_O peak at 533.88 eV was observed in Zn/Fe-LDH@0.5KBC after phosphorus adsorption, further confirming that the adsorption of phosphorus involves interaction with surface -OH hydrogen bonds [67]. For both adsorbents, the Zn 2p spectrum exhibited two characteristic peaks corresponding to Zn 2p_3/2_ and Zn 2p_1/2_ at 1023 eV and 1046 eV, respectively (Figure 8e). Satellite peaks were observed at 1020 eV and 1043 eV beneath the main peaks, which are mainly attributed to the presence of zinc carbonate [68]. After phosphate adsorption, the Zn 2p peaks exhibited a positive shift in binding energy, consistent with the formation of Zn–O–P coordination structures. These results suggest that Zn–OH sites act as Lewis acid sites, facilitating the coordination and complexation of phosphate species [46]. As shown in Figure 8f, two main peaks confirmed the coexistence of Fe^2+^ and Fe^3+^ in both Zn/Fe-LDH and Zn/Fe-LDH@0.5KBC (Figure 6c). For Zn/Fe-LDH@0.5KBC, Fe^2+^ species were associated with Fe 2p _3/2_ at 711.39 eV and Fe 2p_1/2_ at 723.96 eV, while Fe^3+^ species were attributed to higher binding energies at 714.1 eV and 727.6 eV. Although the Fe 2p peaks did not change significantly after adsorption phosphorus, a slight shift toward higher binding energies was observed, further supporting the formation of Fe-O-P bonds [65]. The Fe 2p spectra of Zn/Fe-LDH before and after phosphorus adsorption were similar to those of Zn/Fe-LDH@0.5KBC, but the peak positions of the former exhibited greater shifts. This is mainly due to the higher stability and higher iron binding energy of Zn/Fe-LDH@0.5KBC. These findings are in good agreement with the FTIR results, providing strong evidence that phosphate adsorption proceeds through coordination interactions with the active sites of the adsorbent.

To elucidate the phosphorus adsorption mechanism, a comprehensive analysis combining SEM, XRD, FTIR, and XPS was conducted. As observed in the SEM images, significant morphological changes occurred after phosphorus adsorption, with the disappearance of the original LDH lamellar structures and the formation of plate-like crystalline phases on the surface. These newly formed crystals indicate the occurrence of in situ precipitation processes. This observation is further supported by XRD analysis, where new diffraction peaks corresponding to hopeite (Zn_3_(PO_4_)_2_·4H_2_O) appeared after adsorption, suggesting that phosphate reacted with released Zn^2+^ ions to form insoluble metal phosphate precipitates. FTIR spectra provide additional evidence for phosphate interaction with the adsorbent. After adsorption, characteristic bands attributed to P-O stretching (1019–1105 cm^−1^) and O-P-O bending (568–628 cm^−1^) vibrations emerged, confirming the successful incorporation of phosphate species. Meanwhile, the weakening of -OH and CO_3_^2−^ related peaks suggests that ligand exchange and interlayer anion exchange occurred during adsorption. Further insights from XPS analysis reveal the appearance of P 2p peaks and their shift to lower binding energies, indicating the formation of inner-sphere complexes (Zn-O-P and Fe-O-P). In addition, the decrease in M-O and increase in M-OH components in O 1s spectra confirm the involvement of surface hydroxyl groups in ligand exchange, while the shifts observed in Zn 2p and Fe 2p spectra further support the coordination interaction between phosphate and metal sites.

**Figure 9:**
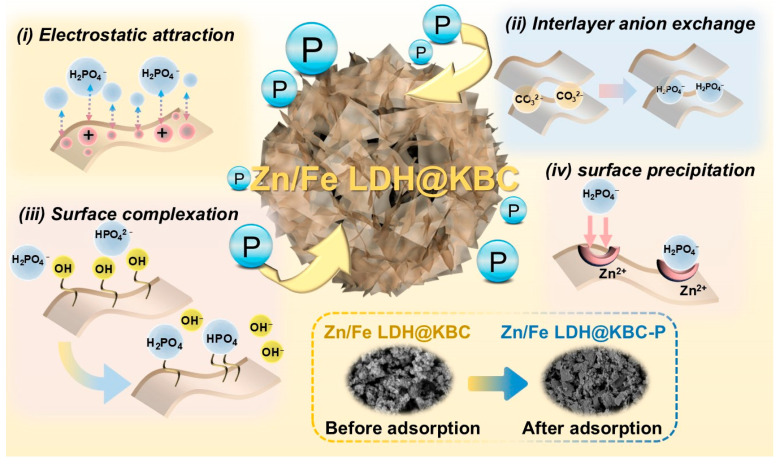
Proposed mechanism of phosphate adsorption on Zn/Fe-LDH@0.5KBC.

Collectively, these results demonstrate that phosphate adsorption onto Zn/Fe-LDH@0.5KBC is governed by the cooperative action of electrostatic attraction, interlayer anion exchange, surface complexation, and surface precipitation. Electrostatic attraction initiates the interaction between negatively charged phosphate species and the positively charged adsorbent surface (Equations (3) and (4)). Subsequently, phosphate ions replace interlayer carbonate via anion exchange (Equation (5)), while ligand exchange between phosphate and surface hydroxyl groups results in the formation of inner-sphere complexes (Equations (6) and (7)). The release of Zn^2+^ under acidic conditions further promotes the in situ formation of insoluble zinc phosphate precipitates (Equation (8)), which are crucial for phosphorus removal. Figure 9 schematically illustrates the detailed mechanism. Importantly, incorporating KBC enhances the dispersion of LDH nanosheets and increases the exposure of active sites, thereby facilitating synergistic adsorption.

Electrostatic attraction:

(3) $$\equiv M-OH+H^{+}\to \equiv M-OH_{2}^{+}$$

(4) $$\equiv M-OH_{2}^{+}+H_{2}PO_{4}^{-}\to \equiv M-OH_{2}^{+}\cdots H_{2}PO_{4}^{-}$$

Interlayer anion exchange:

(5) $$LDH-CO_{3}^{2-}+H_{2}PO_{4}^{-}\to LDH-H_{2}PO_{4}^{-}+CO_{3}^{2-}$$

Surface complexation (ligand exchange):

(6) $$\equiv M-OH+H_{2}PO_{4}^{-}\to \equiv M-H_{2}PO_{4}+OH^{-}$$

(7) $$\equiv M-OH+HPO_{4}^{2-}\to \equiv M-HPO_{4}+2OH^{-}$$

Surface precipitation:

(8) $$3{Zn}^{2+}+2PO_{4}^{3-}+4H_{2}O\to {Zn}_{3}(PO_{4}{)}_{2}\cdot 4H_{2}O↓$$

### 3.4. Potentiality of Phosphorus-Loaded Zn/Fe-LDH@0.5KBC as Fertilizer

**Figure 10:**
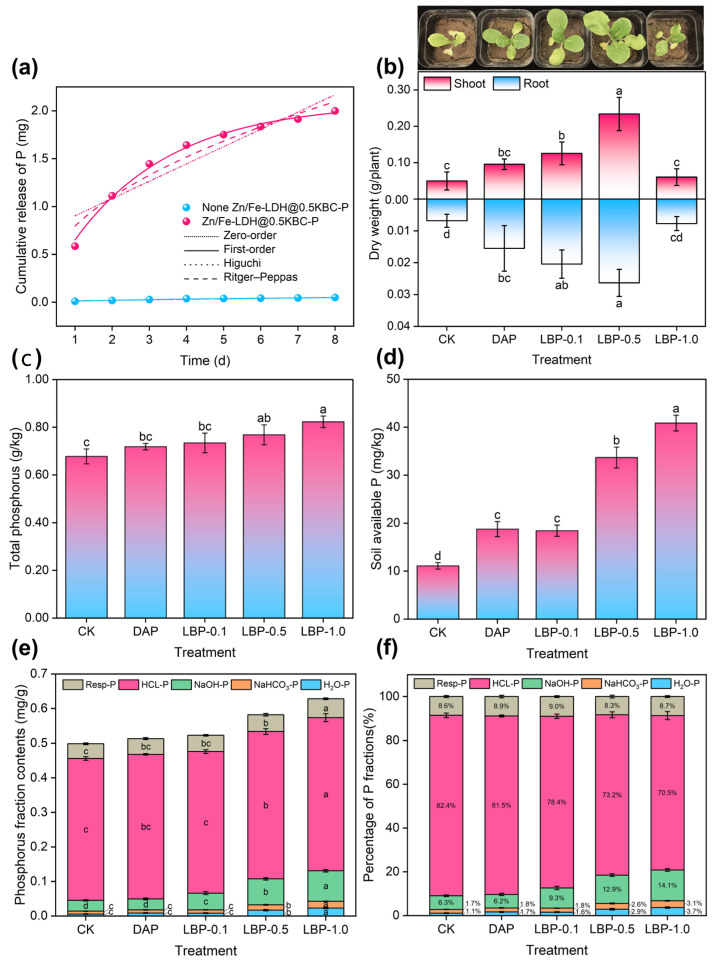
Evaluation of the fertilization performance of Zn/Fe-LDH@0.5KBC-P in fluvo-aquic soil. (**a**) Cumulative phosphorus release and kinetic fitting; (**b**) shoot and root dry biomass of pak choi under different treatments; (**c**) soil available phosphorus; (**d**) soil total phosphorus; (**e**) phosphorus fraction distribution; (**f**) relative proportion of phosphorus fractions. Different lowercase letters indicate significant differences among treatments (*p* < 0.05).

To elucidate the phosphorus release behavior of Zn/Fe-LDH@0.5KBC-P in fluvo-aquic soil, the cumulative release profiles were analyzed using the zero-order, first-order, Higuchi, and Ritger-Peppas kinetic models, with untreated soil (CK) serving as the control. Figure 10a shows that the cumulative phosphorus release increased progressively with leaching time and followed a biphasic release pattern consisting of an initial rapid-release stage followed by a slower and more sustained release phase. The relatively higher release observed on day 1 (0.58 mg) can be attributed to the dissolution of readily available phosphorus located on the external surface or within accessible pores. The kinetic fitting results (Table S6) show that the first-order model provides the best fit for both CK and Zn/Fe-LDH@0.5KBC-P systems (R^2^ > 0.98), suggesting that phosphorus release is primarily governed by a concentration-dependent process. Further analysis using the Ritger–Peppas model reveals that the release exponent (n) for CK is 0.65, indicating a non-Fickian diffusion mechanism involving both diffusion and structural relaxation, whereas the Zn/Fe-LDH@0.5KBC-P system exhibits a lower n value (0.45), indicating a diffusion-dominated release behavior close to Fickian diffusion. This result suggests that incorporating LDH@KBC results in a more regulated, diffusion-governed phosphorus release process [37,69].

Based on the above release characteristics, the fertilization potential of Zn/Fe-LDH@0.5KBC-P was further evaluated through pot experiments using pak choi as the test crop. As shown in Figure 10b, the application of Zn/Fe-LDH@0.5KBC-P significantly increased both shoot and root dry weights compared with CK. Among all treatments, LBP-0.5 exhibited the most pronounced growth-promoting effect, while excessive addition (LBP-1.0) resulted in a slight decline, although still higher than CK. Notably, both LBP-0.1 and LBP-0.5 treatments achieved significantly higher biomass than DAP (*p* < 0.05), indicating the superior fertilization efficiency of the composite material. This trend is consistent with previous studies on controlled-release phosphorus fertilizers [3].

The soil phosphorus analysis (Figure 10c,d) shows that both total phosphorus and available phosphorus contents increased with increasing dosage of Zn/Fe-LDH@0.5KBC-P, indicating an enhanced phosphorus supply capacity in soil. The improved plant growth performance under LBP treatments can be attributed to sustained phosphorus release, which provides a more stable nutrient supply compared with conventional fertilizers. In addition, the layered structure of LDH and the presence of biochar may facilitate gradual phosphorus release and improved nutrient retention in soil, thereby enhancing phosphorus use efficiency [16,17]. To further clarify the transformation of phosphorus forms in soil, sequential extraction was performed. According to the classification system of Cross and Schlesinger, phosphorus can be divided into labile (H_2_O-P and NaHCO_3_-P), moderately labile (NaOH-P), and stable (HCl-P and residual P) fractions [69]. As shown in Figure 10e,f, the application of Zn/Fe-LDH@0.5KBC-P increased the total phosphorus pool, while simultaneously altering its distribution among different fractions. Although HCl-P remained the dominant fraction, its relative proportion decreased from 82.4% (CK) to 70.5%, indicating a relative dilution of stable phosphorus. Meanwhile, the proportion of labile phosphorus increased from 2.8% to 6.8%, and moderately labile phosphorus increased from 6.3% to 14.1%, whereas residual phosphorus remained relatively constant.

These alterations in phosphorus fraction distribution align with the observed release kinetics. The Zn/Fe-LDH@0.5KBC-P system provides sustained phosphorus supply via a diffusion-dominated release process and shifts phosphorus distribution toward more bioavailable and potentially available fractions. In contrast, DAP treatment maintains a high proportion of HCl-P (~81.5%), indicating rapid phosphorus fixation in calcareous soil. Excessive application (LBP-1.0) may be associated with a slight decline in plant growth performance, potentially due to altered soil nutrient balance and possible changes in Zn and Fe bioavailability, which may influence ion uptake processes [70]. Although the composite demonstrated promising performance, the long-term environmental behavior of released Zn and Fe, including their accumulation and ecological consequences, remains to be clarified. In addition, Zn/Fe-LDH@0.5KBC-P provides a practical strategy for integrating controlled phosphorus release with enhanced phosphorus bioavailability, thereby improving fertilizer use efficiency.

## 4. Conclusions

In this study, Zn/Fe-layered double hydroxide-engineered kelp-derived biochar composites were fabricated via a co-precipitation process and subsequently evaluated for phosphate recovery and agricultural reutilization. The incorporation of kelp-derived biochar improved the dispersion of LDH nanosheets, increased the specific surface area, and generated a more open porous structure, thereby enhancing phosphate adsorption. Among the prepared composites, Zn/Fe-LDH@0.5KBC exhibited the highest phosphate adsorption capacity of 132.52 mg P/g, with adsorption behavior mainly governed by chemisorption and well described by the Sips model. The composite maintained effective phosphate uptake under mildly acidic to neutral conditions and showed limited interference from Cl^−^, HCO_3_^−^, and CO_3_^2−^, although NO_3_^−^ markedly suppressed adsorption. Mechanistic analyses based on SEM, XRD, FTIR, and XPS confirmed that phosphate removal resulted from the combined effects of electrostatic attraction, interlayer anion exchange, surface complexation, and metal phosphate precipitation, with in situ zinc phosphate formation playing a major role. The phosphate-loaded Zn/Fe-LDH@0.5KBC further exhibited sustained phosphorus release in soil and improved phosphorus availability. Pot experiments showed that the 0.5% application rate produced the strongest growth-promoting effect on pak choi, while sequential extraction indicated a shift in soil phosphorus toward more labile and moderately labile fractions. These results suggest that Zn/Fe-LDH@KBC can serve as a promising adsorbent–fertilizer material for coupling phosphate recovery from wastewater with soil phosphorus supplementation. Although Zn/Fe-LDH@0.5KBC exhibited the best performance among the KBC loadings investigated in this study (0.5–5.0 g), further optimization using lower KBC loadings (e.g., 0.25 or 0.1 g) remains to be explored. In addition, the long-term phosphorus release behavior, metal leaching risk, and field-scale agronomic performance require further evaluation before practical application.

## Figures and Tables

**Table 1:** The kinetic parameters of phosphorus adsorption onto Zn/Fe-LDH and Zn/Fe-LDH@0.5KBC.

Kinetic Models	Parameters	Adsorbents	
		Zn/Fe-LDH	Zn/Fe-LDH@0.5KBC
q_e-exp_	mg/g	70.86	81.59
PFO	q_e-cal_ (mg/g)	66.41	75.12
	k_1_ (/min)	0.041	0.031
	R^2^	0.961	0.844
PSO	q_e-cal_ (mg/g)	71.37	81.47
	k_2_ (g/mg·min)	7.81 × 10^−4^	6.91 × 10^−4^
	R^2^	0.995	0.991
Elovich	α (mg/g·min)	22.30	89.41
	β (g/mg)	0.0075	0.014
	R^2^	0.952	0.849
Intra particle diffusion	C_1_ (mg/g)	12.32	13.86
	K_d1_ (g/mg·min^−0.5^)	5.61	6.25
	R^2^	0.970	0.985
	C_2_ (mg/g)	48.56	53.36
	K_d1_ (g/mg·min^−0.5^)	1.17	1.58
	R^2^	0.998	0.987
	C_3_ mg/g	65.56	74.84
	K_d1_ (g/mg·min^−0.5^)	0.14	0.18
	R^2^	0.968	0.900

**Table 2:** The isotherm parameters of phosphorus adsorption onto Zn/Fe-LDH and Zn/Fe-LDH@0.5KBC.

Isotherm Models	Parameters	Adsorbents	
		Zn/Fe-LDH	Zn/Fe-LDH@0.5KBC
q_m-exp_	mg P/g	118.01	132.52
Langmuir	Q_max_(mg P/g)	117.17	124.11
	K_L_ (L/mg)	0.132	0.261
	R^2^	0.966	0.924
Freundlich	K_F_ [(mg/g)·(L·mg)^1/n^]	36.76	47.96
	1/n	0.226	0.196
	R^2^	0.922	0.955
Temkin	b_T_ (kJ/mol)	133.59	154.17
	K_T_ (L/g)	3.20	16.45
	R^2^	0.987	0.985
Sips	Q_max_(mg P/g)	134.26	163.49
	Ks (L/mg)	0.198	0.329
	m	1.49	2.13
	R^2^	0.993	0.995

## Data Availability

The original contributions presented in this study are included in the article/Supplementary Materials. Further inquiries can be directed to the corresponding author.

## Supplementary Materials

The following supporting information can be downloaded at: https://www.mdpi.com/article/10.3390/ma19143117/s1, Text S1: Adsorption kinetic and isotherm models; Text S2: Phosphorus release kinetic models; Figure S1: The zeta potential of Zn/Fe-LDH and Zn/Fe-LDH@0.5KBC under different pH conditions; Figure S2: SEM images of Zn/Fe-LDH and Zn/Fe-LDH@0.5KBC at different magnifications; Figure S3: N_2_ adsorption–desorption isotherms and pore size distributions of Zn/Fe-LDH and Zn/Fe-LDH@0.5KBC; Figure S4: SEM images, EDS spectra, and corresponding elemental mapping of Zn/Fe-LDH@0.5KBC before and after phosphate adsorption. The mapped elements include C, O, P, Zn, and Fe; Table S1: Basic physicochemical properties of the soil used in the experiment; Table S2: ICP-OES analysis of Zn and Fe contents and actual Zn/Fe molar ratios of Zn/Fe-LDH@KBC composites prepared with different nominal Zn/Fe precursor ratios; Table S3: Conversion between molar concentrations (mM) and corresponding mass concentrations (mg/L) of the coexisting anions; Table S4: Comparison of adsorption capacity of phosphorus by different LDH biochar composite adsorbents; Table S5: Specific surface area, pore volume, and average pore size of Zn/Fe-LDH and Zn/Fe-LDH@0.5KBC; Table S6: Kinetic parameters of phosphorus release from Zn/Fe-LDH@0.5KBC-P fitted by zero-order, first-order, Ritger-Peppas, and Higuchi models. References [71,72,73,74,75,76,77,78] are cited in the Supplementary Materials.
