# Supplementary material for: Zn/Fe-Layered Double Hydroxide Composites with Kelp-Derived Biochar for Phosphate Recovery and Reutilization as a Slow-Release Fertilizer
Source: Materials (Basel). 2026 Jul 20;19(14):3117. doi: 10.3390/ma19143117 (PMC13414368; doi:10.3390/ma19143117)
Supplement: Supplementary file 1 [file materials-19-03117-s001.zip › materials-4413851-supplementary.pdf]

Supplementary Material

Zn/Fe layered double hydroxide composites with kelp-derived biochar for phosphate recovery and reutilization as a slow-release fertilizer

Authors:

Jin Yang[1,2], Pengcheng Xue[1,2], Lu Zhao[1,2], Yajuan Luo[1,2], Jinfeng Yang[1,2], Mengru Wang[1,2], Guiying Jiang[1,2], Shiliang Liu[1,2]*

Author affiliations:

[1] College of Resources and Environment, Henan Agricultural University, Zhengzhou 450002, China

[2] State Key Laboratory of High-Efficiency Production of Wheat-Maize Double Cropping, Henan Agricultural University, Zhengzhou, Henan 450046, China

Corresponding author:

Name: Shiliang Liu

E-mail: shlliu70@163.com

Permanent address: No.218, Ping An Avenue, Zhengdong New District, Zhengzhou, Henan Agricultural University, 450046, China

Text S1 Adsorption kinetic and isotherm models

Adsorption kinetics models: To explore the adsorption rate and possible rate-controlling mechanisms, several kinetic models were applied, including the pseudo-first-order (PFO), pseudo-second-order (PSO), Elovich, and intraparticle diffusion models.

Pseudo-first-order:

$$q_t = q_e(1 - e^{-k_1 t})$$

Pseudo-second-order:

$$q_t = \frac{k_2 q_e^2 t}{1 + k_2 q_e t}$$

Elovich:

$$q_t = \frac{1}{\beta}\ln(\alpha\beta) + \frac{1}{\beta}\ln(t)$$

Intra-particle-diffusion:

$$q_t = k_d t^{1/2} + C$$

where q_e (mg/g)and q_t (mg/g) are the amount of phosphorus adsorbed at equilibrium and time t, respectively; k_1 (/min), k_2 (g/mg·min) and k_d (g/mg·min$^{-0.5}$) represent the corresponding rate constants for PFO and PSO models; t (min) is the contact time; α is the initial adsorption rate constant and β is the desorption rate constant. C is the intercept.

Adsorption isotherm models: The adsorption isotherm models were used to describe the interaction between phosphate ions and the adsorbent surface at equilibrium. In this study, the Langmuir, Freundlich, Sips, and Temkin isotherm models were employed to analyze the equilibrium adsorption behavior of phosphate onto Zn/Fe-LDH and Zn/Fe-LDH@0.5KBC.

Langmuir:

$$\mathrm{q_e} = \frac{\mathrm{q_m K_L C_e}}{1 + \mathrm{K_L C_e}}$$

Freundlich:

$$\mathrm{q_e} = \mathrm{K_F C_e^{1/n}}$$

Sips:

$$\mathrm{q_e} = \frac{\mathrm{q_m K_s C_e^m}}{1 + \mathrm{K_s C_e^m}}$$

Temkin:

$$q_e = \frac{K_T}{b_T} \ln(K_T C_e)$$

where q_e (mg/g) is the equilibrium adsorption capacity; q_m (mg/g) is the theoretical maximum adsorption capacity; C_e (mg/L) is the equilibrium concentration of phosphate; K_L (L/mg) is the Langmuir constant related to adsorption affinity; K_F [(mg/g)(L/mg)$^{1/n}$] is the Freundlich constant; 1/n represents the adsorption intensity; K_s (L/mg) is the Sips constant; m is the heterogeneity factor; K_T (L/mg) is the Temkin equilibrium binding constant; bT (kJ/mol) is the Temkin isotherm constant.

Text S2 Phosphorus release kinetic models

To investigate how phosphorus is released from P-loaded Zn/Fe-LDH@0.5KBC, we measured the phosphorus concentration in the leachate over time and fit the data to several release kinetic models. We analyzed the phosphorus release kinetics using the zero-order, pseudo-first-order, Elovich, and Higuchi models. The relevant equations are presented below.

Zero-order kinetic model:

$$Q_t = Q_0 + k_0 t$$

First-order kinetic model:

$$Q_t = Q(1 - e^{-k_1 t})$$

Higuchi model:

$$Q_t = k_H t^{1/2}$$

Ritger-Peppas model:

$$Q_t = Q k_{RP} t^n$$

where Q_t (mg) represents the cumulative release amount at time t (d), Q_0 (mg) represents the initial release amount (typically 0), Q (mg) represents the total phosphorus release amount, k_0 (mg/(g d)) is the zero-order release rate constant, k_1 (/d) is the first-order release rate constant, k_H (mg/(g $d^{1/2}$) is the Higuchi diffusion constant related to the diffusion coefficient and matrixporosity, k_{RP} is the Ritger-Peppas release constant, and n is the release exponent indicating the release mechanism.

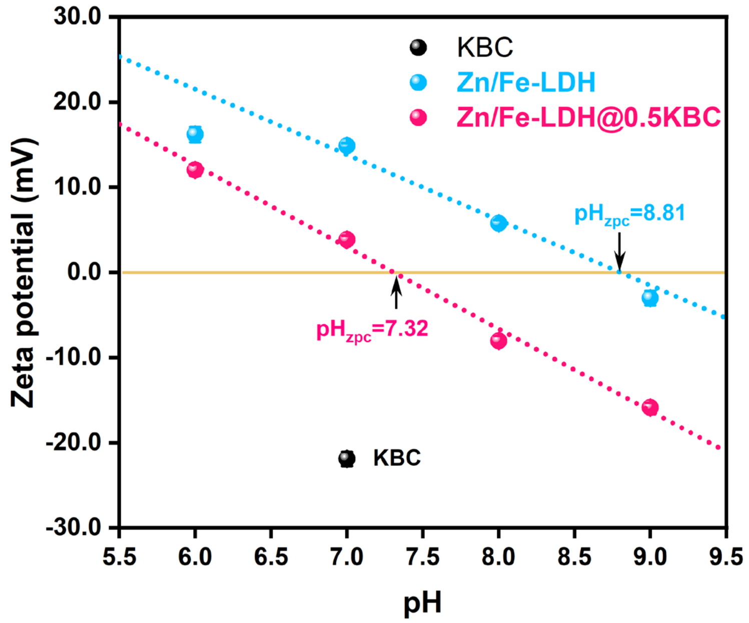

Figure S1 The zeta potential of Zn/Fe-LDH and Zn/Fe-LDH@0.5KBC under different pH conditions.

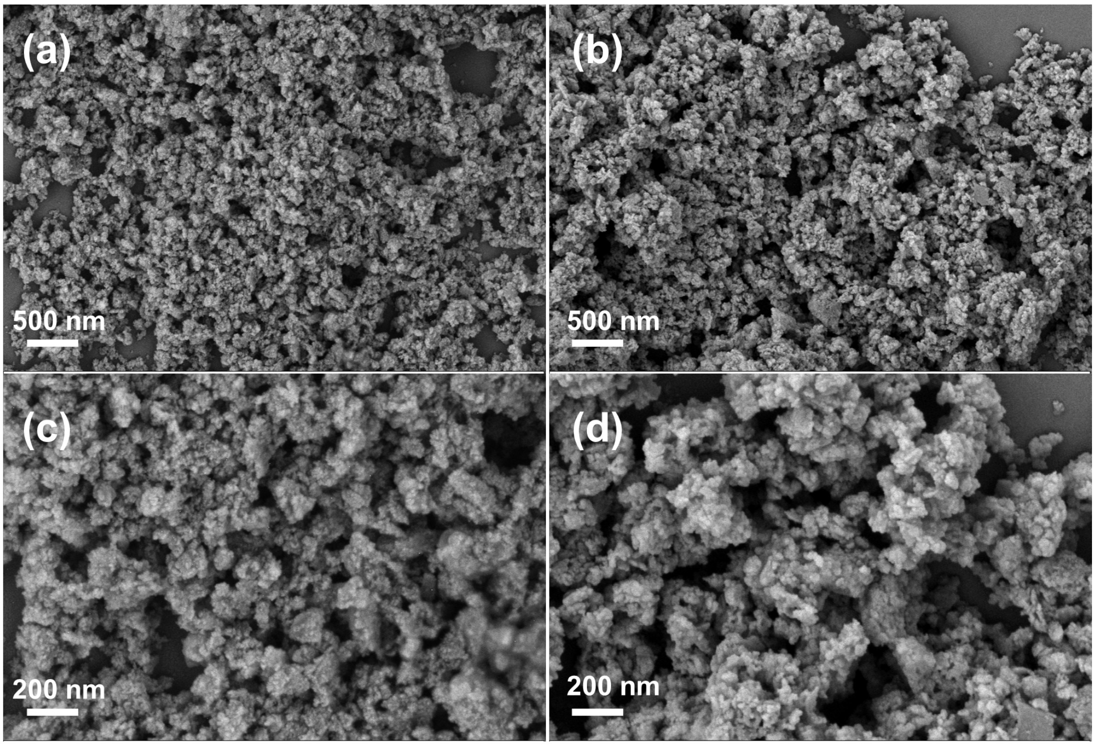

Figure S2 SEM images of Zn/Fe-LDH (a, c) and Zn/Fe-LDH@0.5KBC (b, d) at different magnifications.

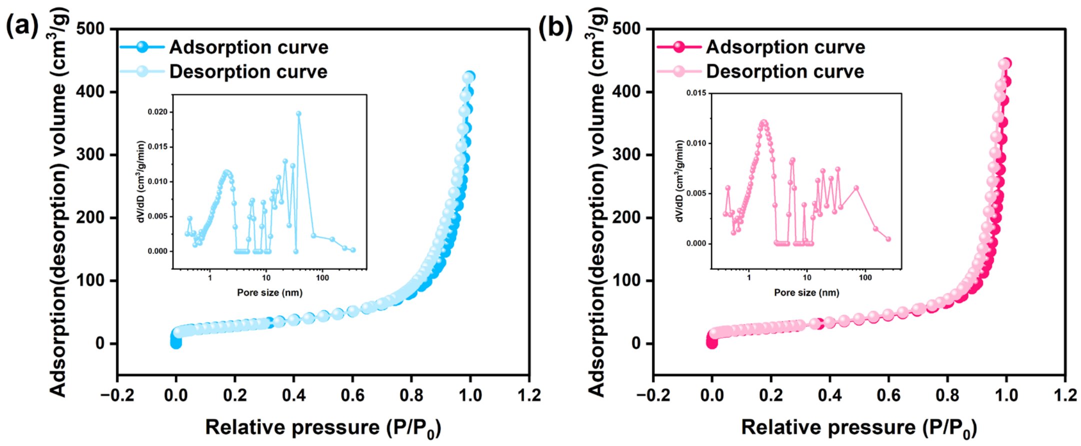

Figure S3 N_2 adsorption-desorption isotherms and pore size distributions of Zn/Fe-LDH (a) and Zn/Fe-LDH@0.5KBC (b).

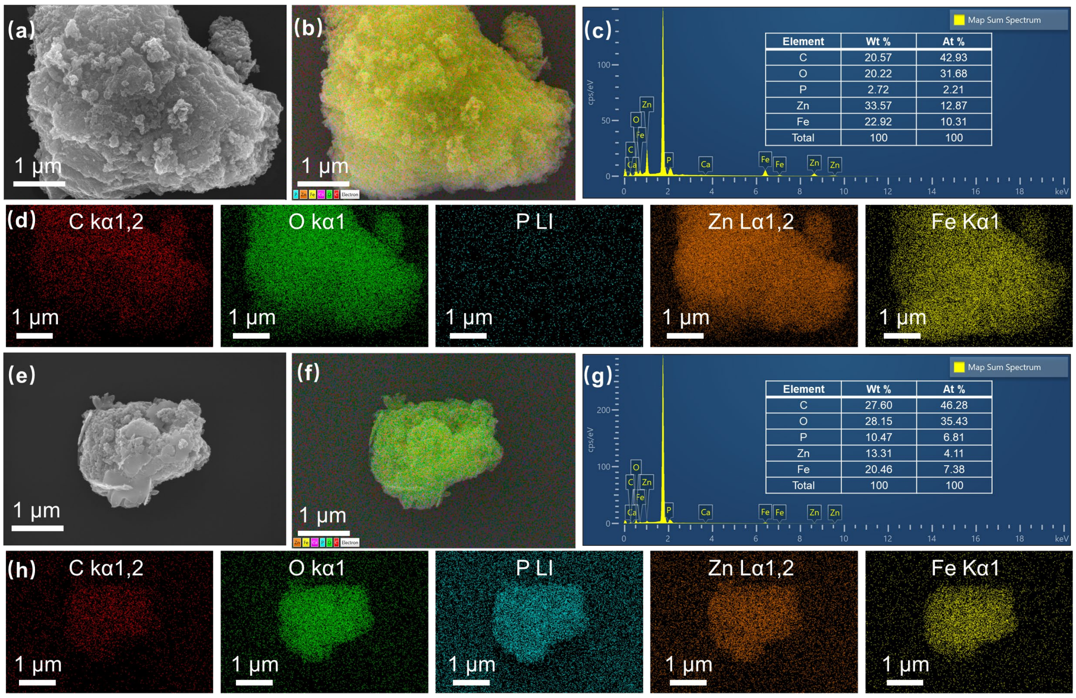

Figure S4 SEM images, EDS spectra, and corresponding elemental mapping of Zn/Fe-LDH@0.5KBC before and after phosphate adsorption. The mapped elements include C, O, P, Zn, and Fe.

Table S1 Basic physicochemical properties of the soil used in the experiment

Parameter	pH	Available phosphorus (mg/kg)	Available potassium (mg/kg)	Organic matter (mg/kg)	Total phosphorus (g/kg)	Alkali-hydrolyzable nitrogen (mg/kg)
	8.72	12.34	89.62	10.07	0.68	42.11

Table S2. ICP-OES analysis of Zn and Fe contents and actual Zn/Fe molar ratios of Zn/Fe-LDH@KBC composites prepared with different nominal Zn/Fe precursor ratios.

**Nominal Zn/Fe precursor ratios**	**Zn (g/kg)**	**Fe (g/kg)**	**Actual Zn/Fe molar ratio**
2:1	462±40	143±66	2.76
3:1	544±52	126±50	3.69
4:1	588±88	86±28	5.84

Table S3. Conversion between molar concentrations (mM) and corresponding mass concentrations (mg/L) of the coexisting anions.

Coexisting anion	Concentration (mM)	Mass concentration (mg/L)
Cl^-	5	177.3
	10	354.5
NO_3^-	5	310
	10	620
HCO_3^-	5	305.1
	10	610.2
CO_3^{2-}	5	300
	10	600
SO_4^{2-}	5	480.3
	10	960.6

Table S4 Comparison of adsorption capacity of phosphorus by different LDH biochar composite adsorbents

Adsorbents	pH	T(℃)	S_{BET} (mg^2/g)	q_{max}(mg P/g)	Reference
Mg/Al LDH biochar composite ($MABC_6$)	3.0	25	26.27	54.93	[71]
Fe/La@BC	-	20	17.82	74.59	[72]
CaLa-BC	5.0	-	47.37	113.04	[73]
$CaLa_2Al_1$-LDH/BC	6.0	25	94.99	168.79	[74]
Zn-Al-LDH/BC	7.2	25	47.28	55.76	[75]
Ni-Fe-LDH/BC	3.0	25	56.1	78.30	[22]
MBC/Mg-Al LDH	5.5	25	-	43.05	[24]
Co-CA	5.0	-	5.57	254.62	[1]
Mg/Al-LDHs biochar	3.0	25	12.25	81.83	[76]
Ra-LDO	-	-	184	132.80	[77]
LDHCF@CWB	-	25	78.8	37.4	[78]
Zn/Fe-LDH@0.5KBC	3.0	25	122.13	163.49	This study

Table S5 Specific surface area, pore volume, and average pore size of Zn/Fe-LDH and Zn/Fe-LDH@0.5KBC

Materials	Specific surface area (m^2/g)	Pore volume (cm^3/g)	Average pore size (nm)
Zn/Fe-LDH	94.41	0.64	27.25
Zn/Fe-LDH@0.5KBC	122.13	0.71	23.55

Table S6 Kinetic parameters of phosphorus release from Zn/Fe-LDH@0.5KBC-P fitted by zero-order, first-order, Ritger-Peppas, and Higuchi models

Materials	Model	Parameter	Value	R^2
None Zn/Fe-LDH@0.5KBC-P	Zero-order	k_0	0.005	0.958
	First-order	k_1	0.200	0.980
	Higuchi	k_H	0.0106	0.906
	Ritger-Peppas	k_{RP}	0.0126	0.950
		n	0.650	
Zn/Fe-LDH@0.5KBC-P	Zero-order	k_0	0.224	0.836
	First-order	k_1	0.375	0.994
	Higuchi	k_H	0.362	0.836
	Ritger-Peppas	k_{RP}	0.795	0.933
		n	0.450	

Reference

[1] Zhou, Z.; Luo, D.; Zhang, X.; Wang, C. Sustainable phosphorus recovery from wastewater by layered double hydroxide/biochar composites for potential agricultural application. Ind. Crops Prod. 2025, 224, 120422.

[22] Yang, F.; Zhang, S.; Sun, Y.; Tsang, D.C.W.; Cheng, K.; Ok, Y.S. Assembling biochar with various layered double hydroxides for enhancement of phosphorus recovery. J. Hazard. Mater. 2019, 365, 665–673.

[24] Ihsanullah, I.; Almanassra, I.W.; Abushawish, A. Macadamia nut shell biochar/Mg-Al LDH composite: A sustainable solution for highly effective phosphate ion removal from water. J. Water Process Eng. 2024, 67, 106164.

[71] Zhou, A.; Wu, Q.; Zhang, H.; Liu, J.; Wang, C.; Chi, D.; Zhou, H. RSM-optimized Mg/Al-LDH biochar composite for enhanced phosphorus removal: Insights into interlayer structure evolution and adsorption mechanism. J. Environ. Manag. 2026, 401, 128837.

[72] Nie, W.; Zhang, X.; Luo, X.; Xie, L.; Zhu, Y.; Tang, A.; Liang, D. Efficient phosphate adsorption from real industrial wastewater using Fe/La-impregnated biochar. J. Environ. Chem. Eng. 2025, 13, 117967.

[73] Nan, H.; Luo, D.; Zhang, X.; Wang, J.; Wang, C. Ca/La layered double hydroxide functionalized biochar: Preparation, characterization, phosphate adsorption mechanism, and post-evaluation of phosphorus availability. Mater. Today Commun. 2025, 48, 113296.

[74] Zhang, Q.; Wu, X.; Xiong, R.; Wu, B.; Yv, C.; Li, Q.; Wang, L.; Zhang, Z.; Yang, X.; Li, S. Synergistically regulating the microstructure of CaLaAl layered double hydroxide/biochar through pore confinement and defect design for efficient phosphorus recovery from water. J. Environ. Chem. Eng. 2026, 14, 121036.

[75] Zhang, J.; Huang, W.; Yang, D.; Xiang, J.; Chen, Y. Removal and recovery of phosphorus from secondary effluent using layered double hydroxide-biochar composites. Sci. Total Environ. 2022, 844, 156802.

[76] Li, R.; Wang, J.J.; Zhou, B.; Awasthi, M.K.; Ali, A.; Zhang, Z.; Gaston, L.A.; Lahori, A.H.; Mahar, A. Enhancing phosphate adsorption by Mg/Al layered double hydroxide functionalized biochar with different Mg/Al ratios. Sci. Total Environ. 2016, 559, 121–129.

[77] Zhang, Z.; Yan, L.; Yu, H.; Yan, T.; Li, X. Adsorption of phosphate from aqueous solution

by vegetable biochar/layered double oxides: Fast removal and mechanistic studies. Bioresour. Technol. 2019, 284, 65–71.
[78] Shin, J.; Rho, H.; Cho, Y.; Son, C.; Kwak, J.; Kim, S.; Ki, S.; Kim, H.-J.; Lee, Y.-G.; Park, Y.; Lee, S.-H.; Chon, K. Functionalization of coffee waste biochars with Ca/Fe layered double hydroxides for enhanced removal and selectivity of phosphate ions: Mechanisms and reusability. J. Water Process Eng. 2025, 76, 108123.
